# Evaluation of serum interleukin-12 and interleukin-4 as potential biomarkers for the diagnosis of major depressive disorder

**DOI:** 10.1038/s41598-024-51932-9

**Published:** 2024-01-18

**Authors:** Nisat Sarmin, A. S. M. Roknuzzaman, Tashfiya Zaman Mouree, Md. Rabiul Islam, Zobaer Al Mahmud

**Affiliations:** 1 Department of Clinical Pharmacy and Pharmacology, Faculty of Pharmacy, University of Dhaka, Dhaka, 1000 Bangladesh; 2 Department of Pharmacy, University of Asia Pacific, Dhaka, 1205 Bangladesh; 3 Department of Pharmaceutical Technology, Faculty of Pharmacy, University of Dhaka, Dhaka, 1000 Bangladesh; 4 School of Pharmacy, BRAC University, KHA 224, Progati Sarani, Merul Badda, Dhaka, 1212 Bangladesh

**Keywords:** Neuroscience, Biogeochemistry, Biomarkers, Risk factors

## Abstract

Recently, scientists have focused on pro-inflammatory cytokines and immunological dysregulation in major depressive disorder (MDD). Some research suggests pro-inflammatory cytokines' role in MDD development, whereas anti-inflammatory studies are sparse. There is no systematic investigation of Bangladeshi MDD patients' pro- and anti-inflammatory cytokines. This study examines the blood levels of IL-12 and IL-4 in Bangladeshi patients and healthy controls (HCs) to determine the diagnostic accuracy of these cytokines to identify MDD patients from those without MDD. A total of 110 people with MDD from the department of psychiatry of a teaching hospital in Dhaka and 107 HCs from Dhaka participated in this case–control study. Depression and illness severity were gauged using DSM-5 criteria and Ham-D scores. Commercially marketed ELISA kits were used in accordance with manufacturer guidelines to measure the levels of IL-12 and IL-4 in peripheral blood, allowing a comparison of the patient and control groups. In comparison to HCs, MDD patients (5333.00 ± 307.40 pg/ml) showed noticeably higher levels of IL-12 than in HCs (2331.00 ± 207.40 pg/ml). The increased levels were positively correlated with Ham-D scores (male: r = 0.351, p < 0.050; female: r = 0.389, p < 0.050), suggesting a possible relationship to disease progression. Additionally, compared to HCs (272.81 ± 23.94 pg/ml), MDD patients had significantly higher peripheral blood levels of IL-4 (876.35 ± 66.73 pg/ml) (p < 0.001). Also, there was a positive correlation between IL-4 serum levels and Ham-D scores (male: r = 0.361, p < 0.050; female: r = 0.398, p < 0.050). Therefore, we observed increased levels of these serum cytokines and their association with the severity of depression. The results of this study demonstrate the possibility of IL-12 and IL-4 blood levels as distinct markers capable of differentiating between MDD patients and HCs, possibly acting as markers of MDD susceptibility. To ascertain the diagnostic effectiveness of these two cytokines, more research is necessary.

## Introduction

Major depressive disorder (MDD) is a serious neuropsychiatric ailment that affects a substantial number of the world's population^1^. A continuous state of low mood and low self-esteem characterizes MDD. It is now widely acknowledged that MDD is a significant factor in mental disability worldwide. It is prevalent and substantially impacts various aspects of individuals' lives-routine, work, education, family, and social interactions-thus posing a considerable public health challenge. There is still a lack of thorough understanding of the disease's underlying pathophysiology, diagnostic techniques, and viable treatments, although it has a high prevalence and noteworthy deleterious effects^2,3^. Traditional explanations for the pathophysiology of anxiety and depression-related illnesses entail the disruption of monoaminergic pathways, which results in changes in neurotransmission and a disparity between excitatory and inhibitory signaling^4–6^. On the other hand, there is an increasing interest in investigating the possibility that immunomodulatory factors, such as cytokines and chemokines, have a role in the development of MDD^7–14^. Recent advancements in the field of psychoneuroimmunology have led to the discovery of new knowledge regarding the reciprocal linkages that exist between conditions such as anxiety and depression and chronic, low-grade inflammation. In the development of MDD, it has been proposed that peripheral inflammatory responses play a role^15–20^. Studies in which pro-inflammatory cytokines like IL-2 and INF-γ were injected into healthy controls (HCs) or animals showed that these cytokines can evoke symptoms that are comparable to those of MDD^14,19–22^. In addition to this, the consumption of antidepressant drugs was associated with a perceptible lowering in the severity of depression^21^.

On the other hand, those who are battling depression have been shown to exhibit increased acute inflammatory responses, which is a phenomenon that has been validated by previous research^21,23,24^. The results of this study highlight the potential role of inflammatory cytokines not just in the early phases of MDD, but also in the symptoms of MDD that persist over time. A number of studies have shown that people who have been diagnosed with MDD have higher levels of chemokines and cytokines that are pro-inflammatory in their bodies and a notable correlation exists between illness severity and the levels of these cytokines^10–12,25–33^. The MCP-1, IL-1, TNF-α, CRP, and IL-6 are some of them. However, there has been a dearth of substantial exploration into the potential links between peripheral anti-inflammatory cytokines like IL-4 and IL-10 and MDD. Additionally, thorough investigations into the relationship between inflammation levels, the efficacy of anti-inflammatory interventions, and their impact on MDD severity have been notably absent, both within Bangladesh and globally. This study observed significant reductions in MCP-1, BDNF, IL-7, and IL-10 levels in individuals of Bangladeshi descent diagnosed with MDD than controls^34–36^.While studies have demonstrated associations between MDD severity and concentrations of pro-inflammatory cytokines such as IL-2, IL-6, and MCP-1 in the blood of Bangladeshi patients^37–39^, research on concentration of anti-inflammatory cytokines like IL-4 in MDD patients from the same population is currently lacking.

Dendritic cells, monocytes, macrophages, and antigen-presenting cells are all responsible for the production of the pro-inflammatory cytokine IL-12. The heterodimeric form of IL-12 is the one that occurs naturally, and it requires cooperation from both growing pathogenic bacteria and B cells^40^. It makes a considerable contribution to cell-mediated and innate immunity by supporting type 1 T helper (Th1) responses^41,42^. IL-12 is responsible for the development of naive T cells into effector Th1 cells. It then stimulates NK cells and T cells to boost IFN-γ production, which is a cytokine that is beneficial for phagocytic activity and the commencement of an inflammatory response.^41–45^. The initial stimulation of Th1 immune responses by IL-12 is necessary for the later modulation of Th2 immune responses, which is regulated by IL-12 and serves to reduce inflammatory processes. The putative relationship between changed IL-12 levels and the pathophysiology of MDD has been the subject of a number of important lines of inquiry and found responsible for wide range of inflammatory effects^46^. Patients diagnosed with MDD have been shown to have higher serum IL-12 levels in contrast to individuals who do not have the disorder, according to a number of case–control studies and meta-analyses^14,21,26,29,46,47^.In addition, it has been reported that individuals who have been diagnosed with MDD and who display higher levels of IL-12 at baseline demonstrate a considerable drop after undergoing therapy with antidepressant or anti-inflammatory drugs^10,14,21,46^. Researchers have put out a large number of ideas^48^ on the mechanism by which IL-12 contributes to the etiology of depressive-like symptoms and behaviors seen in MDD. An important finding relates to the activation of Indoleamine 2,3-dioxygenase (IDO), an enzyme related with the metabolism of tryptophan, by pro-inflammatory cytokines such as IL-1 and IL-12. An important amino acid known as tryptophan is a precursor to the neurotransmitter serotonin, which plays a critical role in the regulation of mood. Because IL-12 encourages the breakdown of serotonin, increased levels of IL-12 have been linked to decreased serotonin levels. As a consequence of this, one may have symptoms that are comparable to those of depression.

In contrast, IL-4 is an essential cytokine that possesses anti-inflammatory properties. It does this by inhibiting the production and transmission of pro-inflammatory cytokines such as IL-1, IL-6, and TNF-α ^49,50^. Recent studies have shed light on the function that IL-4 plays in activating M2 macrophages, which contributes to the anti-inflammatory effects of IL-4 by successfully lowering pro-inflammatory responses. However, there is ongoing discussion over the possibility of a connection between the anti-inflammatory cytokine IL-4 and the degree of severity of MDD. Although some researchers have found a link between lower levels of IL-4 in the blood and a higher risk of MDD, other studies have suggested that persons with MDD typically demonstrate greater levels of IL-4 in their blood opposed to those who do not have MDD. This is because people with MDD are more likely to have inflammation in their bodies. In spite of the fact that a number of studies have found a connection between reduced levels of IL-4 in the blood and the degree of severity of MDD, a pattern that contradicts this finding has emerged. One study that exemplifies this concept is one that was carried out in 2007 by Kim et al. and looked at the differences between HCs and those who had been diagnosed with MDD. According to the results of this experiment, those who had been diagnosed with MDD had a significant reduction in the levels of IL-4 production in their bodies. In contrast, the research carried out by Chen et al. (2017) and Kohler et al. (2017) did not find any significant differences in the levels of IL-4 found in the blood of patients diagnosed with MDD and a control cohort made up of people who did not have any mental health disorders. Additional study is required to investigate the association between the severity of MDD and IL-4^25,26,29^ in order to help bridge the knowledge gap that currently exists. The completion of this research is absolutely necessary in order to close the current learning gap.

The diagnosis of MDD depends on doctors evaluating subjective symptoms that patients report, frequently based on patients or family members' recollections. Along with the results of the Ham-D scale, this diagnosis method also takes into account the criteria listed in the DSM-5^51–54^. However, it's critical to recognize that the method's lack of objectivity and low specificity may result in misinterpretations in about 40% of instances^10,30,51^. Given the facts so far, conducting research and developing a blood-based biomarker for MDD that can objectively predict disease development and is less intrusive, affordable, and simple to use is imperative. The potential of cytokines as blood-based biomarkers for many illnesses, including MDD, has recently been investigated by numerous research groups^10,30,49^. The ability to distinguish between the blood levels of IL-8 and adiponectin in Bangladeshi MDD patients and HCs has significant diagnostic potential^55,56^. According to Karlovic et al.'s (2013) research, calculating blood BDNF levels had a reasonable diagnostic accuracy for identifying people with MDD. However, only two distinct investigations have looked at the diagnostic efficacy of the anti-inflammatory cytokine IL-4 in distinguishing HCs from MDD patients, with conflicting findings^10,49,57^. More research is required to examine the diagnostic effectiveness of IL-4 in MDD thoroughly. The effectiveness of using blood levels of IL-12 to distinguish between those with MDD and those without the disorder is still up for debate. With a good discriminative AUC value of 0.871^58^, Nahar et al. (2022) established the ability of peripheral IL-12 levels to identify people with MDD in the Bangladeshi community successfully. However, a different study by Xu et al. (2023) on Chinese MDD patients revealed that IL-12 had poor diagnostic performance, with an AUC value of 0.660^10^. Given this situation, it is anticipated that serum concentration of IL-12, and IL-4 may serve as blood-derived biomarkers for MDD that can be used for both diagnosis and prognosis. These biomarkers may be used to predict and recognize MDD in its early stages and to evaluate the effectiveness of antidepressant medications. IL-12, a pro-inflammatory cytokine, and IL-4, an anti-inflammatory cytokine, were measured in peripheral blood in this study in Bangladeshi patients with MDD and HCs. The primary objective of this research was to evaluate the association between the severity of MDD in patients from Bangladesh and the concentrations of the anti-inflammatory cytokine IL-4 as well as the inflammatory mediator IL-12 that were found in their peripheral blood and to determine whether these cytokines may be used as risk assessment markers for depression.

## Methods and materials

### Study subjects

Participants in this case–control study included a total of 110 individuals diagnosed with MDD and 107 HCs. The study recruited MDD patients from the department of psychiatry of a teaching hospital in Dhaka, Bangladesh. The control group comprised individuals from various parts of Dhaka city, meticulously matched with patients regarding age, sex, and BMI. Patients were diagnosed by a psychiatrist following DSM-5 criteria, with the same psychiatrist evaluating control records. The severity of the condition was determined using the widely acknowledged and reliable Ham-D, which comprises 21 questions, but the scoring relies on the first 17 items. Scores range from 0 to ≥ 23, reflecting symptom severity^59^. Physical and neurological assessments were conducted to identify coexisting conditions. We included participants who had no history of liver or kidney failure and did not use any antidepressant or antipsychotic medication affecting the targeted cytokine levels for at least two weeks preceding the blood sample being taken. The study excluded those with concurrent psychiatric problems, including other AXIS I disorders and autoimmune disorders, mental impairments, alcohol or substance abuse, major organic disorders, significant obesity, or infectious diseases. Socio-demographic data were collected using pre-designed questionnaires. Heights, weights, and BMIs were measured. The study adhered to the Declaration of Helsinki guidelines, and participants were informed about the study's purpose and provided consent before participation.

### Collection of blood samples

A 5 ml blood was drawn from each participant's cephalic vein. Samples were coagulated at ambient temperature for an hour before undergoing 15 min of centrifugation at 3000 rpm to extract serum samples. Extracted serum samples were placed in Eppendorf and stored at − 80 °C for analysis.

### Estimation of serum IL-12 and IL-4

Serum levels of IL-12 and IL-4 were estimated using enzyme-linked immunosorbent assays (ELISA tests) for both patients and controls. These assays were performed on both groups. In order to accomplish this goal, human IL-12 ELISA kit EZ-set and human IL-4 ELISA kit EZ-set, both of which are available on the market from Boster Bio in Pleasanton, California, USA, were employed, and the instructions that the manufacturer supplied were followed.

### Statistical analysis

We analyzed the data using GraphPad Prism (version 5.0b) and SPSS (version 24.0). In order to provide a concise summary of the clinical and socio-demographic features contained within the study, descriptive statistics were applied. In order to provide a clear picture, these statistics were provided as the mean ± standard error of the mean (SEM). The two-tailed student's t-test was utilized in order to determine whether or not there were statistically significant differences in the mean values of age, sex, BMI, and any other relevant factors that differentiated the patients in the hospital group from the control group. Graphical displays allowed for the realization of visual representations of contrasts between several subject groups in a study. The use of a two-tailed unpaired student's t-test was implemented for the purpose of analyzing the differences in cytokine levels that were found in the blood of persons who suffered from MDD as opposed to those who served as the control group. Pearson's correlation analysis was used to evaluate any potential correlations that may exist between the levels of cytokines and the Ham-D scores, which were used to quantify the severity of the disorder. This study was conducted with the goal of illuminating any potential connections that may exist between the two variables. The ROC curve analysis was used in the ongoing project to differentiate between individuals diagnosed with MDD and their HCs. This differentiation was accomplished by analyzing the levels of IL-4 and IL-12. Following standard procedures allowed for the computation of diagnostic accuracy. When the p-value that was obtained was lower than 0.05, statistical significance was determined to have been achieved.

### Ethical considerations

The research ethics committee, University of Asia Pacific, Dhaka, Bangladesh, granted approval for the study protocol (Ref: UAP/REC/2022/S-1). The participants were provided with a comprehensive overview of the objectives of this study, and their informed consent was acquired in written form. The study was conducted in accordance with the principles outlined in the Helsinki Declaration.

## Results

### Socio-demographic details of the study participants

Table 1 presents an overview of the socio-demographic characteristics of the participants in the study. All the participants were similar in terms of their age, sex, and BMI. The mean age of the patient group diagnosed with MDD and the group of HCs was 31.91 ± 0.92 and 32.74 ± 0.94 years, respectively. The two groups had comparable distributions regarding male and female ratios, with a p-value of 0.060. The mean BMI of the patient group (24.70 ± 0.46) was found to be similar to that of the HCs (22.48 ± 0.40), as indicated by a p-value of 0.550. Furthermore, it is noteworthy that there was no statistically significant disparity in terms of other socio-demographic parameters. However, the groups were different based on their family history of MDD.

**Table 1:** Socio‐demographic profile of the study population.

Parameters	MDD patients n (%)	Healthy controls n (%)	p value
Age in years	31.91 ± 0.92	32.74 ± 0.94	0.920
18 − 24	31 (28.18)	19 (17.76)	
25 − 39	56 (50.91)	62 (57.94)	
46 − 60	23 (20.91)	26 (24.30)	
Sex			0.062
Male	46 (41.82)	42 (39.25)	
Female	64 (58.18)	65 (60.75)	
Marital status			0.279
Married	66 (60.0)	59 (55.14)	
Unmarried	44 (40.0)	48 (44.86)	
BMI (kg/m^2^)	24.70 ± 0.46	22.48 ± 0.40	0.550
Below 18.5 (CED)	6 (5.45)	13 (12.15)	
18.5–25.0 (normal)	60 (54.55)	68 (63.55)	
Above 25.0 (obese)	44 (40.00)	25 (23.35)	
Education level			0.437
Illiterate	8 (7.27)	5 (4.67)	
Primary level	26 (23.64)	45 (42.06)	
Secondary level	53 (48.18)	51 (47.66)	
Graduate and above	23 (20.91)	6 (5.61)	
Occupation			0.057
Business	7 (6.36)	18 (16.82)	
Service	12 (10.91)	19 (17.76)	
Unemployed	12 (10.91)	25 (23.36)	
Student	5 (4.55)	2 (1.87)	
Others	74 (67.27)	43 (40.19)	
Economic status			0.143
High	12 (10.91)	14 (13.08)	
Medium	26 (23.64)	31 (28.97)	
Low	72 (65.45)	62 (57.95)	
Smoking history			0.871
Nonsmoker	85 (77.27)	84 (78.50)	
Smoker	25 (22.73)	23 (21.50)	
Residence area			0.283
Rural	66 (60.00)	77 (71.96)	
Urban	44 (40.00)	30 (28.04)	
Family history of MDD			< 0.001
Yes	26 (23.64)	2 (1.87)	
No	84 (76.36)	105 (98.13)	

*BMI* body mass index, *CED* chronic energy deficiency, *MDD* major depressive disorder, *n* number.

### Clinical characteristics and laboratory results

**Figure 1:**
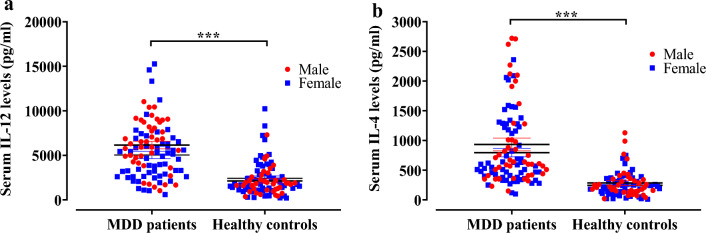
Comparison of serum cytokines in MDD patients and healthy controls (**a**) serum IL-12 levels and (**b**) serum IL-4 levels. Data were analyzed by two-tailed unpaired t test to evaluate the level of significance between mean difference between two groups. [***] represents p-value < 0.001.

Table 2 displays the clinical features of the study population. In the group of patients diagnosed with MDD, the mean Ham-D scores and DSM-5 values were found to be 17.47 ± 0.43 and 7.15 ± 0.14, respectively. In the present case–control investigation, MDD patients had significantly elevated blood levels of IL-12 (5333.00 ± 307.40 pg/ml) in comparison to the HCs (2331.00 ± 207.40 pg/ml) (Table 2, Fig. 1), with statistical significance seen at a p-value of less than 0.001. The study observed that individuals diagnosed with MDD had a substantially greater level of IL-4 in their peripheral serum (876.35 ± 66.73 pg/ml) compared to the HCs (272.81 ± 23.94 pg/ml) (Table 2 and Fig. 1), with a p < 0.001. A further noteworthy discovery was that there is no significant difference in blood levels of IL-4 between male and female patients diagnosed with MDD (p > 0.05).

**Table 2:** Clinical profile and laboratory findings of the study population.

Parameters	MDD patients (n = 110) mean ± SEM	Healthy controls (n = 107) mean ± SEM	p value
Ham‐D score	17.47 ± 0.43	3.14 ± 0.25	< 0.001
Male	16.67 ± 0.67	2.42 ± 0.33	
Female	18.05 ± 0.54	3.63 ± 0.37	
Serum IL‐12 (pg/ml)	5333.00 ± 307.40	2331.00 ± 207.40	< 0.001
Male	5551.00 ± 401.10	2126.00 ± 229.50	
Female	4728.00 ± 442.70	2661.00 ± 437.00	
Serum IL‐4 (pg/ml)	876.34 ± 66.72	272.81 ± 23.94	< 0.001
Male	986.60 ± 124.20	284.90 ± 38.80	
Female	795.50 ± 70.47	245.10 ± 24.65	

*MDD* major depressive disorder, *SEM* standard error mean, *Ham-D* 17-item Hamilton depression rating scale, *IL-12* interleukin-12, *IL-4* interleukin-4.

**Figure 2:**
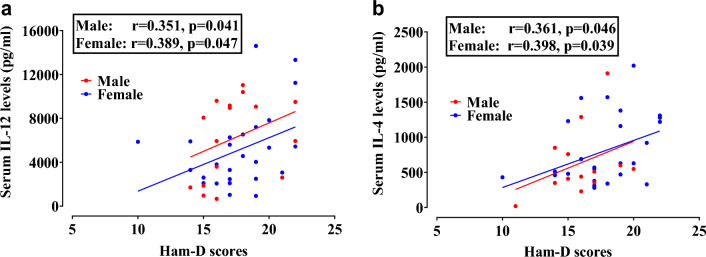
Scatter diagram of Ham-D scores versus (**a**) serum IL-12 levels and (**b**) serum IL-4 levels in the patient group. Pearson correlation analysis was performed to find out the potential association between serum cytokine levels and Ham-D scores of MDD. Both the male and female MDD patients exhibited moderately strong association between IL-12 or IL-4 serum levels and Ham-D scores.

To investigate the potential link between the severity of MDD and elevated blood concentrations of IL-12 and IL-4, a Pearson's correlation analysis was conducted. The results of this study unveiled a statistically significant positive correlation (p < 0.05) between the serum level of IL-12 and the Ham-D scores. This observation suggests that heightened IL-12 levels in the bloodstream are intertwined with an escalation in disease severity. Notably, the strength of association between IL-12 serum concentration and MDD severity was characterized as moderate, with a Pearson correlation coefficient (male: r = 0.351, p < 0.050; female: r = 0.389, p < 0.050). Likewise, the application of Pearson's correlation assessment identified a statistically significant positive relationship between Ham-D scores and peripheral levels of IL-4 within the group of MDD patients. Furthermore, this connection was found to be relatively robust, with a correlation coefficient (male: r = 0.361, p < 0.050; female: r = 0.398, p < 0.050), signifying a substantial correlation between peripheral serum IL-4 levels and the severity of the disorder (Fig. 2). We also performed correlation analysis between serum cytokines and Ham-D scores in control group. However, we didn’t observe any significant association between cytokine levels and Ham-D scores (supplementary Fig. 1).

### Receiver operating characteristic curve analysis

**Figure 3:**
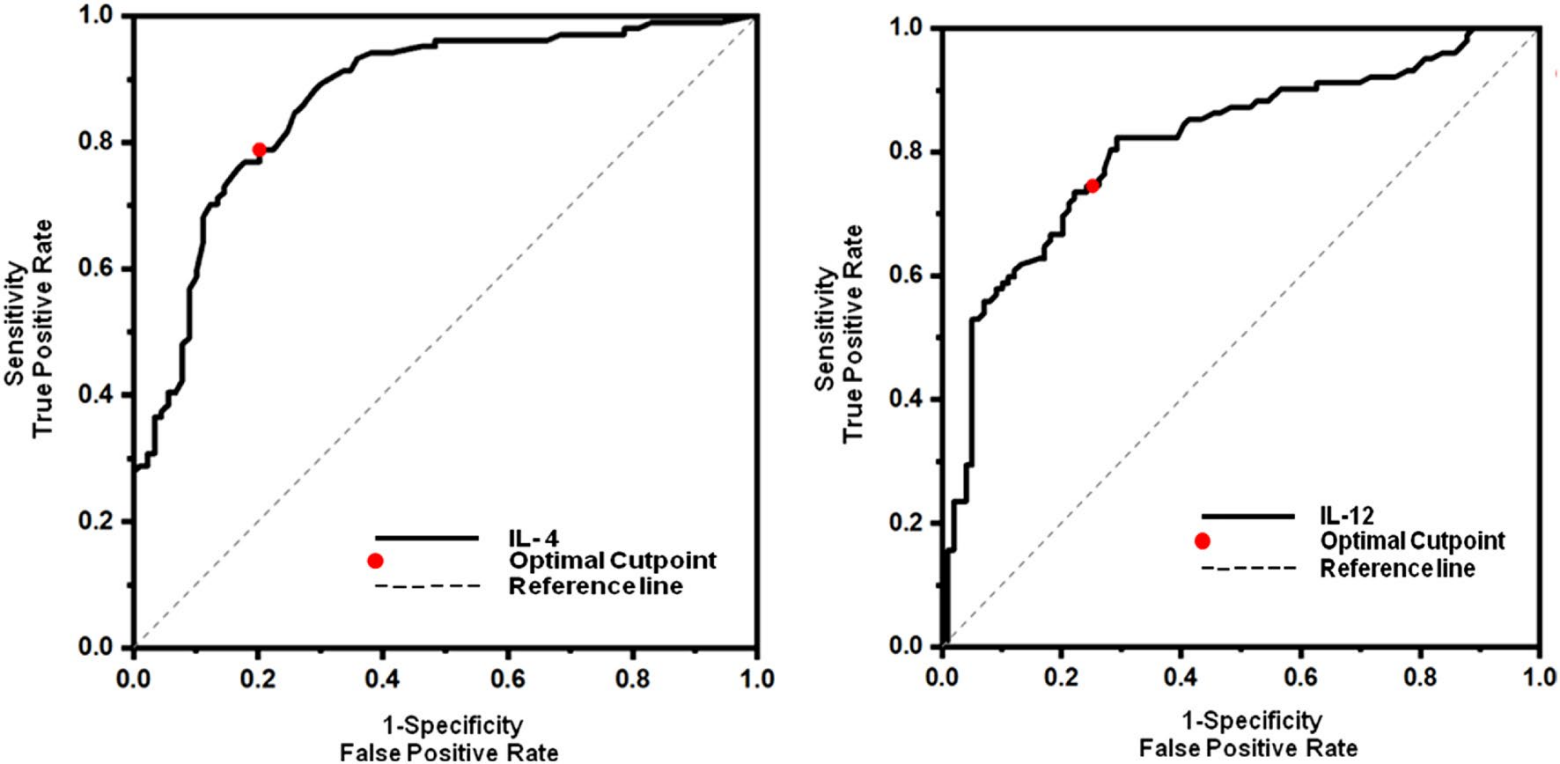
Receiver operating characteristic (ROC) curve for serum IL-12 and IL-4 levels in discriminating MDD patients from healthy controls.

The findings pertaining to the investigation of the ROC curve for serum IL-12 and IL-4 concentrations have been displayed in Table 3 and Fig. 3. Based on the study conducted, it was determined that the established cut-off value for IL-12 was 2817.00 pg/ml. The sensitivity and specificity values were found to be 74.50% and 74.75%, respectively. In the case of IL-4, the cut-off point value was determined to be 375.00 pg/ml, with corresponding sensitivity and specificity rates of 78.84% and 79.78%, respectively. The AUC for IL-12 was found to be 0.812, with a confidence range ranging from 0.750 to 0.870 (p < 0.001). Similarly, the AUC for IL-4 was determined to be 0.870 (p < 0.001), with a confidence interval spanning from 0.820 to 0.920.

**Table 3:** Receiver operating characteristic curve analysis for measuring serum levels of IL-4 and IL-12 as diagnostic biomarkers for MDD.

Cytokine	Cut-off value (pg/ml)	AUC	95% CI		Sensitivity (%)	Specificity (%)	PPV (%)	NPV (%)	p value
			Lower limit	Upper limit					
IL-12	2,817.00	0.812	0.752	0.870	74.50	74.75	75.25	74.00	< 0.001
IL-4	375.00	0.870	0.820	0.920	78.84	79.78	67.40	83.00	< 0.001

*AUC* area under the curve, *CI* confidence interval, *PPV* positive predictive value, *NPV* negative predictive value, *IL-12* interleukin-12, *IL-4* interleukin-4.

## Discussion

Our case–control study revealed a significant elevation in blood levels of IL-12 among individuals with MDD compared to HCs, aligning with findings from multiple prior investigations^10,21,46,47^. The MDD patients exhibited approximately 2.3 times higher blood IL-12 levels than HCs. Interestingly, there was no statistically significant difference in IL-12 blood concentrations between male and female MDD patients (p > 0.05)^47^. In contrast, MDD patients displayed noticeably heightened peripheral serum levels of IL-4 when compared to HCs. This observation concurs with results from other studies^49,60^ that also reported substantial increases in IL-4 serum levels in MDD patients compared to controls. Conversely, Kim et al. (2007) found significantly lower IL-4 blood levels in MDD patients (94.14 ± 56.45 pg/ml) relative to controls (267.72 ± 113.52 pg/ml)^29^. Likewise, Sutcigil et al. (2007) documented significantly reduced IL-4 serum levels in MDD patients (3.43 ± 0.51 pg/ml) in comparison to HCs (7.80 ± 1.75 pg/ml)^47^.

When comparing MDD patients to the HCs, we observed significantly elevated blood levels of IL-12 in the MDD patients. Pearson correlation analysis revealed a substantial positive association between the severity of MDD and increased IL-12 blood levels, which was also reflected in Ham-D scores. These findings were consistent with another case–control study conducted in Bangladesh^58^ and globally^10,14,29^. These findings imply a potential link between the pathophysiology of MDD and IL-12 serum levels within the Bangladeshi population. Nevertheless, it remains uncertain whether the heightened IL-12 serum level acts as an etiological factor in depression formation or is an outcome of excessive inflammatory responses following depressive syndromes. The potential association between MDD's etiology and peripheral IL-12 levels suggests the possibility of innovative treatment strategies targeting IL-12-mediated pro-inflammatory behaviors associated with MDD upon further research.

In comparison, the compensatory rise in anti-inflammatory cytokine concentrations, which is a response to increasing levels of pro-inflammatory cytokine, could be the cause of elevated IL-4 levels among the MDD patient group. Another study reveals that persons with MDD trigger the compensatory immunoregulatory response system (CIRS) to counteract the overreactive inflammatory response system (IRS), thereby maintaining immunological balance^61^. In patients suffering from MDD, there is an increase in levels of pro-inflammatory cytokines such as IL-6, TNF-α, IL-8, IL-12, and IL-1. Anti-inflammatory cytokines such as IL-4 are produced in large quantities to combat the exaggerated inflammatory response that is caused by the presence of pro-inflammatory cytokines. These anti-inflammatory cytokines exert their regulatory function by either blocking the generation of pro-inflammatory cytokines or generating phenotypes associated with M2 macrophages^49^. In line with this, the results of our investigation showed that the IRS was also active in Bangladeshi persons who had been diagnosed with MDD. This is evidenced by the fact that patients with MDD have 2.3 times higher amounts of the pro-inflammatory cytokine IL-12 in their blood than the HCs does. In addition, activation of the CIRS was detected, which was demonstrated by a threefold rise in the anti-inflammatory cytokine IL-4 among MDD patients in comparison to HCs. This compensatory increase in IL-4 serum levels among MDD patients, as a response to the worldwide rise in pro-inflammatory cytokines, indicates that IL-4 levels in blood evaluation, as opposed to the tracking of pro-inflammatory cytokines, could serve as an appealing diagnostic and prognostic biomarker for MDD. It is important to note that this compensatory elevation in IL-4 serum levels among MDD patients occurred as a response to the global rise in pro-inflammatory cytokines.

The data presented in Table 2 reveals a stark contrast in Ham-D scores and cytokine levels between male and female patients with MDD. As indicated by the Ham-D scores, the mean score for female MDD patients is 18.05 ± 0.54, which is higher than the mean score for male patients (16.67 ± 0.67). This discrepancy suggests that females may experience more severe depressive symptoms. This is consistent with the results obtained from multiple studies and the global prevalence of MDD by sex, thus strengthening the validity of this claim^36,38,62,63^. Significant differences can be observed in the levels of serum IL-12 and IL-4 cytokines between male and female patients diagnosed with MDD. Male patients diagnosed with MDD demonstrate notably elevated concentrations of serum IL-12 (5,551.00 ± 401.10 pg/ml) in comparison to their female counterparts (4,728.00 ± 442.70 pg/ml). Likewise, when it comes to serum IL-4, male patients with MDD exhibit considerably higher concentrations (986.60 ± 124.20 pg/ml) than their female counterparts (795.50 ± 70.47 pg/ml). This finding implies a possible association between reduced concentrations of the anti-inflammatory cytokine IL-4 and heightened intensity of depressive symptoms, as measured by the Ham-D score, among females compared with males. Hormonal fluctuations may account for the observed gender differences in cytokine levels and Ham-D scores, given that estrogen has been demonstrated to regulate the immune response^64^. Furthermore, the observed disparities may be influenced by psychosocial factors, genetic predispositions, and the interplay between neurotransmitter systems and inflammatory pathways. The potential influence of environmental stressors is noteworthy, considering the significant disparities in lifestyle between males and females in Bangladesh^65,66^. Research has documented comparable findings, wherein studies have identified gender-specific differences in cytokine profiles among individuals diagnosed with MDD^67^. The intricate and multifaceted relationship between inflammatory markers and depressive symptoms requires further study in order to clarify the fundamental mechanisms at play and to determine the potential ramifications for tailored therapeutic approaches.

A substantial and positive association was found in this study between IL-12 and IL-4 blood levels and the Ham-D scores of MDD patients. This suggested that both cytokines are linked with illness severity. The ROC analysis found that measuring the IL-4 serum concentration had a very strong predictive value in distinguishing MDD patients from controls, with an AUC value of 0.870. This was determined by the results of the ROC analysis. The measurement of the IL-4 serum level demonstrated a sensitivity of 78.84% and a specificity of 79.78% when the cutoff point was set at 375.00 pg/ml. These diagnostic performance indicators show that IL-4 has promising diagnostic value, and as a result, it might be utilized for the risk assessment of individuals suffering from MDD. Xu et al. (2023) showed that the IL-4 peripheral level had an AUC of 0.675, which is lower than the clinically acceptable value of 0.700 and, as a result, is not clinically beneficial in differentiating patients from HCs^10^. Our findings contradict those reported by Xu et al., who found that the AUC was lower than the clinically acceptable value. Because our result and the one published by Xu et al. (2023) on the diagnostic effectiveness of IL-4 are incompatible, it is imperative that more research be conducted that involves the recruitment of a larger sample of individuals.

Due to the lack of practical and objective diagnostic tools, the diagnosis of MDD is now based on the patients' own subjective experiences, the behavioral observations of family members or friends, and an appraisal of the patients' mental state. It is widely acknowledged that the subjective nature of this method is a crucial factor in the erroneous diagnosis and unsuccessful treatment of MDD. As a result, the creation of innovative diagnostic tools is absolutely necessary if we are going to improve the accuracy of our treatment of this ailment. It is considered that cytokine biomarkers that are derived from blood have the potential to differentiate between people who have MDD and HCs^10,30,68^. In addition, the use of these biomarkers has a number of benefits in comparison to the more invasive and expensive conventional procedures. The assessment of cytokines is less invasive, more cost-effective, simpler to administer, and it provides an objective diagnostic method^69–71^. In addition, biomarkers that are based on cytokines can help with the early detection of sickness, which paves the way for timely treatment. But as of right now, there isn't a reliable peripheral biomarker that doesn't include any invasive procedures that can diagnose MDD^57^. Studies that investigate the diagnostic significance of IL-4 for MDD are few and far between. This is despite the fact that a number of research groups have previously investigated the possibility of using a variety of cytokines, including IL-2, IL-8, IL-10, IL-12, TNF-α, BDNF, MCP-1, and MCP-4 as blood-based biomarkers for the diagnosis and prognosis of MDD^10,52^. The utility of IL-4 as a biomarker of diagnosis for MDD and bipolar disorder has only been examined by two research teams so far. However, their findings were contradictory; Xu et al. (2023) came to the conclusion that IL-4 had a poor diagnostic performance^10^, but Lu et al. (2023) indicated that IL-4 blood levels had a high clinical predictability^49^. This difference, in addition to contradicting results regarding the diagnostic accuracy of IL-4, necessitates the completion of more study.

Here we aimed to investigate the practicability of using IL-4 as a possible diagnostic biomarker that might be obtained from blood samples in order to identify and differentiate MDD patients from HCs. This is the first study of its sort, and it looked at the levels of IL-4 in the blood of people of Bangladeshi ancestry who had MDD. We hypothesized that there might be a connection between the levels of IL-4 in the periphery of the body and the development of MDD since we found that people with MDD had much higher levels of IL-4 in their blood than HCs did. Xu et al. (2023) found that patients with MDD in China had blood IL-4 levels that were twice as high as those seen in HCs (16.4 ± 71.84 pg/ml)^10^, while our research found that patients with MDD in Bangladesh had IL-4 levels that were three times higher than those found in controls (272.81 ± 23.94 pg/ml). The conclusions of Xu and colleagues are invalidated by this discrepancy. The significant differences in IL-4 levels, which were 27.4 times higher in Bangladeshi MDD patients compared to Chinese MDD patients, and in the fold increase in MDD patients relative to controls, which were 16.5 times higher in Bangladeshi controls compared to Chinese control subjects, may have been caused by differences in the study subjects, biases in the detection methods, or racial and ethnic differences between the Chinese and Bangladeshi populations. The changes in IL-4 blood levels and disparities in fold rise between MDD patients and HCs in the Chinese and Bangladeshi populations could be related to differences in way of life, eating habits, xenobiotic exposure, and other factors between these two study groups. These variations were found in both populations studied. For instance, in comparison to Bangladeshis, people from China tend to have a lifestyle that is more conducive to sitting around all day.

Growing clinical evidence indicates that sertraline exhibits some anti-inflammatory effects in MDD patients by reducing levels of inflammatory cytokines. Additionally, celecoxib might serve as an adjuvant antidepressant, as demonstrated by Abbasi et al. (2012), who found that celecoxib treatment significantly decreased depressive symptoms, attributed to its downregulation of IL-6 concentrations. Furthermore, multiple clinical studies indicated better outcomes in reducing depressive symptoms when antidepressants like sertraline and fluoxetine were administered alongside COX-2 inhibitors celecoxib or TNF-α antagonists etanercept or infliximab. This was in contrast to the outcomes of single antidepressant therapy^72–74^. These findings suggest the potential utilization of anti-inflammatory medications as complementary treatment options for severe depression. Based on these considerations, there might be a potential therapeutic strategy for the development of adjuvant treatment for MDD by targeting the inflammatory pathways mediated by pro- and anti-inflammatory cytokines, including IL-12 and IL-4, which needs further investigation.

## Strengths and limitations

The case–control research approach that was employed for this work has several inherent drawbacks, the most notable of which is an inability to capture treatment responses as well as changes in peripheral IL-12 and IL-4 levels among MDD patients throughout the study. In addition, if the inquiry is restricted to only IL-12 and IL-4, only a subset of the complete pathophysiology of MDD may be covered. It would be beneficial to incorporate the measurement of other parameters within the identical sample and facility setting in order to get more complete information. This would take place within the framework of the study. In addition, the influence of dietary habits and physical exercise or any environmental stressors on the parameters that were evaluated was not addressed, which highlights the requirement for cohort studies in a population that is comparable and contains a variety of cytokines that are either pro- or anti-inflammatory.

In spite of these drawbacks, the present investigation does have some essential positives. There have only been a few research studies carried out all over the world to explore the effects of anti-inflammatory cytokines on individuals suffering from MDD, and the results have been equivocal. In addition, the research sample was purposefully selected to be both varied and homogenous, which improved the generalizability of the findings and increased their reliability. In addition, sex-specific examinations of blood IL-12 and IL-4 levels in MDD patients were carried out. The results of these investigations provided more nuanced knowledge of potential sex variations in these biomarkers.

## Conclusion

As per we know this is the first scientific study reporting the potential of IL-4 as an indicator for the risk assessment of MDD patient of Bangladeshi origin. This case–control study investigating the potential of IL-12 and IL-4 cytokines as blood-based biomarkers for MDD would have several clinical implications. This study will contribute to our understanding of the role of IL-12 and IL-4 in the pathophysiology of MDD. The identification IL-12 and IL-4 as potential risk assessment indicators could lead to improved early detection, personalized management approaches, and better patient outcomes. As accurate early detection of a complicated disease is instrumental to clinical intervention and effective disease management, use of these cytokines as risk assessors will identify the individuals with higher risk for developing MDD at an early stage. This early detection of risk for MDD will enable the concerned individual to start the management and/therapeutic interventions at an earlier stage leading to a greater protection from the morbidity and mental disability from MDD.

### Supplementary Information

Supplementary Information.

## Data Availability

The data supporting the present study findings are obtainable from corresponding authors upon reasonable request.
