# Supplementary material for: Evaluation of serum interleukin-12 and interleukin-4 as potential biomarkers for the diagnosis of major depressive disorder
Source: Sci Rep. 2024 Jan 18;14:1652. doi: 10.1038/s41598-024-51932-9 (PMC10796357; doi:10.1038/s41598-024-51932-9)
Supplement: Supplementary file 1 — Supplementary Information. [file 41598_2024_51932_MOESM1_ESM.docx]

**Supplementary Fig 1.** Scatter diagram of Ham-D scores vs (a) serum IL-12 levels and (b) serum IL-4 levels in the healthy controls group. Pearson correlation analysis was conducted to find out any potential association between Ham-D scores and cytokine serum levels. Serum IL-12 and IL-4 levels did not show any correlation with Ham-D scores of healthy control subjects.
